# Objectively measured physical activity and cardiac biomarkers: A cross sectional population based study in older men

**DOI:** 10.1016/j.ijcard.2017.11.003

**Published:** 2018-03-01

**Authors:** Tessa J. Parsons, Claudio Sartini, Paul Welsh, Naveed Sattar, Sarah Ash, Lucy T. Lennon, S. Goya Wannamethee, I-Min Lee, Peter H. Whincup, Barbara J. Jefferis

**Affiliations:** a UCL Department of Primary Care & Population Health, United Kingdom; b Institute of Cardiovascular and Medical Sciences, BHF Glasgow Cardiovascular Research Centre, United Kingdom; c Brigham and Women's Hospital, Harvard Medical School, United States; d Population Health Research Institute, St George's University of London, United Kingdom

**Keywords:** Physical activity, Sedentary behaviour, N-terminal pro-brain natriuretic peptide, Troponin T, Biomarkers, Men

## Abstract

**Background:**

N-terminal pro-brain natriuretic peptide (NT-proBNP) and high sensitivity Troponin T (hsTnT) are markers of cardiac injury used in diagnosis of heart failure and myocardial infarction respectively, and associated with increased risk of cardiovascular disease. Since physical activity is protective against cardiovascular disease and heart failure, we investigated whether higher levels of physical activity, and less sedentary behaviour were associated with lower NT-proBNP and hsTnT.

**Methods and results:**

Cross sectional study of 1130 men, age 70–91 years, from the British Regional Heart Study, measured in 2010–2012. Fasting blood samples were analysed for NT-proBNP and hsTnT. Physical activity and sedentary behaviour were measured using ActiGraph GT3X accelerometers. Relationships between activity and NT-proBNP or hsTnT were non-linear; biomarker levels were lower with higher total activity, steps, moderate/vigorous activity and light activity only at low to moderate levels of activity. For example, for each additional 10 min of moderate/vigorous activity, NT-proBNP was lower by 35.7% (95% CI − 47.9, − 23.6) and hsTnT by 8.4% (95% CI -11.1, − 5.6), in men who undertook < 25 or 50 min of moderate/vigorous activity per day respectively. Biomarker levels increased linearly with increasing sedentary behaviour, but not independently of moderate/vigorous activity.

**Conclusion:**

Associations between biomarkers and moderate/vigorous activity (and between hsTnT and light activity) were independent of sedentary behaviour, suggesting activity is driving the relationships. In these older men with concomitantly low levels of physical activity, activity may be more important in protecting against cardiac health deterioration in less active individuals, although reverse causality might be operating.

## 1 Introduction

N-terminal pro-brain natriuretic peptide (NT-proBNP) and high sensitivity Troponin T (hsTnT) are routinely measured clinical biomarkers. Although the biologically active natriuretic peptides have protective diuretic, natriuretic, and metabolic effects, elevated NT-proBNP is used in the diagnosis and monitoring of heart failure [1], and is a strong marker of ventricular stretch and cardiac overload. In contrast hsTnT is a marker of myocardial ischaemia used in the diagnosis of myocardial infarction [2]. In observational studies both are associated with increased risk of cardiovascular disease and all-cause mortality, over and above other routine risk factors, even in apparently healthy people [3], [4], [5], [6]. Importantly, both biomarkers have been shown to predict cardiovascular events [6], and it has been recently suggested that NTproBNP assessment could integrate heart failure into CVD primary prevention [7].

Two recent systematic reviews have reported that physical activity is protective against incident heart failure [8], [9], and a number of studies suggest that the benefits are greater at or confined to higher levels of physical activity [9]. Physical activity improves many of the established heart failure risk factors such as hypertension, obesity, diabetes, smoking and coronary artery disease [10], and may also reduce the age-related decline in cardiac structure and function [11]. Sedentary behaviour, defined as sitting or reclining as distinct from a low level of physical activity has been little investigated in relation to heart failure but there is some evidence that sedentary behaviour may increase heart failure risk independently of physical activity [12].

Few studies have investigated relationships between physical activity and NT-proBNP or hsTnT, and findings are inconsistent. A study of older adults (≥ 65 years) found that more time spent in accelerometer measured walking was associated with lower levels of NT-proBNP and hsTnT [13], whilst of two studies using self-reported measures of physical activity, one found that participants doing more activity were less likely to experience an increase in NT-proBNP or hsTnT level during follow up [14], and the other found no associations between physical activity and biomarkers [15]. Self-report data for physical activity has limitations, particularly for light activity and sedentary behaviour and in older adults who have lower levels of physical activity and are more sedentary [16]. We investigated associations between both objectively measured physical activity and sedentary behaviour and biomarkers NT-proBNP and hsTnT, in a sample of community dwelling older men.

## 2 Methods

### 2.1 Sample

The British Regional Heart Study is a population-based cohort study following 7735 men recruited from primary care practices in 24 British towns in 1978–80. In 2010–2012, 3137 survivors were invited to (i) a physical examination, which included providing a fasting blood sample and (ii) wear a physical activity monitor. 1722 men (55%) attended, 1603 of whom provided a blood sample with NT-proBNP and hsTnT levels analysed. We excluded 272 men with a reported diagnosis of heart attack, heart failure, or stroke, leaving 1331 men with biomarker data. The National Research Ethics Service (NRES) Committee London provided ethical approval. Participants provided informed written consent to the investigation in accordance with the Declaration of Helsinki.

### 2.2 Objective physical activity assessment

Men wore the GT3X accelerometer (Actigraph, Pensacola, Florida) over the right hip for 7 days, during waking hours, removing it for swimming or bathing. Counts per minute (CPM) were calculated from movements registering on the vertical axis. Data were processed using standard methods [17]. Non-wear time was excluded using the R package “Physical Activity” [18]. Valid wear days were defined by convention as ≥ 600 min wear time, and participants with ≥ 3 valid days were included in analyses. Each minute of activity was categorised using widely used intensity threshold values of counts per minute developed for older adults: < 100 for sedentary behaviour (< 1.5 MET), 100–1040 for light activity (1.5–3 MET) and > 1040 for moderate/vigorous activity,(≥ 3 MET) [19].

### 2.3 Cardiac injury biomarkers

Men were requested to fast for at least 6 h and instructed to drink only water during this time and take medications as usual. Blood samples were collected between 08:00 h and 19:00 h, centrifuged and separated the same day and stored on site at − 20 °C until they were transferred (within 2 weeks) to a central freezer storage location at − 70 °C. Samples were subsequently transferred to a central laboratory on dry ice and thawed immediately prior to analysis. NT-proBNP and hsTnT were measured using electrochemiluminescence immunoassays, performed on a Roche Elecsys 2010 automated platform (Roche Diagnostics, Burgess Hill, UK). The NT-proBNP and hsTnT assays have lower detection limits of 5 pg/mL and 3 ng/L, respectively [20], [21]. For both assays, results below the limit of detection were assigned a value of 50% of the functional limit of detection (2.5 pg/mL for NT-proBNP and 1.5 ng/L for hsTnT). Assays were performed using the manufacturer's calibrators and quality controls. NT-proBNP and hsTnT had assay coefficients of variation of 6.5% and 4.5% for the low control and 3.8% and 9.1% for the high control, respectively.

### 2.4 Covariates

Men completed a questionnaire including information about: current cigarette smoking, alcohol consumption, living alone, current use of antihypertensives, statins and anticoagulants, ever receiving a doctor diagnosis of heart attack, heart failure, and stroke (with symptoms lasting > 24 h). Social class was based on longest held occupation at study entry (1978–80) and categorised as manual and non-manual. Region of residence (1978–80) was grouped into Scotland, North, Midlands and South of England. Body mass index (BMI, kg/m^2^) was calculated from height (Harpenden stadiometer) and weight in light indoor clothing (Tanita body composition analyser (BC-418) or Tanita scales if the participant had a pacemaker or defibrillator).

### 2.5 Statistical methods

We excluded men reporting a diagnosis of heart attack, heart failure, or stroke (with symptoms lasting > 24 h) from analyses. Descriptive statistics for social and demographic characteristics, physical activity and sedentary behaviour, were calculated by quartile of NT-proBNP and hsTnT levels. The distributions of NT-proBNP and hsTnT were skewed and therefore transformed using natural logarithm.

We investigated associations between a number of physical activity measures and NT-proBNP or hsTnT. Physical activity measures included total activity counts per day, steps per day, minutes per day of moderate and vigorous activity, light activity and sedentary behaviour. We first used generalised additive models (GAMs) to investigate whether the relationships between each physical activity measure and NT-proBNP or hsTnT were nonlinear, using the “mgcv” package in R (version 3.0.3). GAM is a non-parametric model that does not specify the shape of any nonlinearity. To quantify associations between physical activity and NT-proBNP/hsTnT we used linear regression models (Stata version 13), and where associations were nonlinear we used the Stata function “mkspline”. Mkspline allows the relationship to be estimated as a piecewise linear function (a function composed of linear segments), joining at knots. The position of the knot(s) (turning point of the function) was estimated from the GAM plots. All models were adjusted for average accelerometer wear time (minutes/day), season of accelerometer wear (warm, May–September or cold, October–April), hour of blood sampling, age, region of residence, social class, living alone, smoking status and alcohol. For ease of interpretation we present percentage differences in biomarker levels for each additional 10,000 counts of total activity, 1000 steps, 30 min of sedentary behaviour or light activity and 10 min of moderate/vigorous activity per day. To evaluate the independence of associations of activity intensities, models were mutually adjusted; (i) moderate/vigorous activity and sedentary behaviour and (ii) moderate/vigorous activity and light activity in the same model. Sedentary behaviour and light activity were not included in the same model due to collinearity (*r* = − 0.62). We further adjusted all models for BMI to investigate whether BMI modified relationships between physical activity/sedentary behaviour and the biomarkers. To explore the potential of undiagnosed heart failure influencing findings we repeated regression models after excluding men with NTproBNP > 400 pg/L. We conducted a post hoc analysis to investigate whether baseline levels of biomarkers and their association with physical activity differed by hypertensive status.

## 3 Results

Of 1331 men with biomarker levels available, 45 had an undetectable level of NT-proBNP (allocated value 2.5 pg/L) and 38 men had undetectable hsTnT (allocated value 1.5 ng/L). 1130 men had data for biomarkers, physical activity and all covariates. Men spent on average 614, 200 and 41 min per day in sedentary behaviour, light activity and moderate/vigorous activity respectively, had a mean of 5004 steps and 167,397 accelerometer counts per day (Table 1). Eighty percent of men had 7 days of accelerometer data and 96% had ≥ 5 days of data. Men with higher levels of NT-proBNP or hsTnT were older, more likely to live alone, take statins or anti-hypertensive medication, consume less alcohol, have lower levels of physical activity and higher levels of sedentary behaviour (Table 1, Table 2). Men with higher levels of hsTnT were also more likely to have diabetes (Table 2).

**Table 1:** Characteristics of 1331 men without pre-existing CVD or heart failure, by quartile of NTproBNP. Figures are mean (SD) unless stated otherwise.

	Quartile of NTproBNP (pg/mL)				*P* (trend)	All men	*N*
	2.5–<68	≥ 68–<134	≥ 134–<312	≥ 312–8322			
	Median = 39	Median = 98	Median = 198	Median = 619			
	(*n* = 362)^a^	(*n* = 357)^a^	(*n* = 329)^a^	(*n* = 283)^a^			
Age (years)	76.6 (3.8)	77.8 (4.1)	79.4 (4.7)	80.7 (5.2)	< 0.0001	78.5 (4.7)	1331
Manual social class,% (*n*)	49 (176)	46 (163)	44 (143)	43 (120)	0.4	46 (602)	1317
Lives alone,% (*n*)	15 (54)	19 (66)	23 (76)	22 (61)	0.03	20 (257)	1311
Smoker,% (*n*)	3.4 (12)	3.4 (12)	4.7 (15)	3.3 (9)	0.8	3.7 (48)	1308
Taking statins,% (*n*)	44 (159)	40 (144)	44 (146)	47 (134)	0.4	44 (583)	1331
Taking anti-hypertensives,% (*n*)	47 (170)	50 (178)	53 (175)	65 (184)	< 0.0001	53 (707)	1331
Taking anticoagulants,% (*n*)	0.8 (3)	2.8 (10)	2.7 (9)	19.8 (56)	< 0.0001	5.9 (78)	1331
Diabetic,% (*n*)	12 (45)	15 (52)	11 (36)	17 (49)	0.1	14 (182)	1331
Alcohol (units per week)	7.5 (8.3)	5.9 (7.1)	6.1 (7.4)	5.9 (7.5)	0.01	6.4 (7.6)	1296
BMI (kg/m^2^)	27.0 (3.7)	27.0 (3.9)	27.1 (3.6)	26.8 (3.9)	0.6	27.0 (3.8)	1319
Accelerometer wear time (min/day)	863 (70)	854 (69)	855 (61)	847 (64)	0.008	855 (67)	1184
Total activity (counts per day)	194,742 (102,400)	180,323 (102,384)	149,464 (93,570)	136,170 (90,943)	< 0.0001	167,397 (100,542)	1184
Steps/day	5813 (2775)	5351 (2883)	4550 (2672)	4040 (2556)	< 0.0001	5004 (2815)	1184
% time spent sedentary	69.9 (9.1)	70.8 (9.2)	73.2 (8.6)	74.7 (9.9)	< 0.0001	72 (9)	1184
% time light activity	24.4 (6.9)	24.1 (6.9)	22.7 (6.5)	21.6 (7.7)	< 0.0001	23 (7)	1184
% time moderate/vigorous activity	5.7 (4.0)	5.2 (3.6)	4.1 (3.5)	3.7 (3.5)	< 0.0001	4.7 (3.8)	1184
Sedentary behaviour (min/day)	603 (87)	602 (81)	625 (79)	631 (81)	< 0.0001	614 (83)	1184
Light activity (min/day)	211 (34)	207 (65)	194 (59)	185 (71)	< 0.0001	200 (65)	1184
Moderate/vigorous activity (min/day)	50 (34)	45 (32)	35 (32)	32 (31)	< 0.0001	41 (33)	1184

Pearson chi square test used for all categorical variables except smoking for which Fisher's exact test was used.

BMI, body mass index.

NT-proBNP, N-terminal pro-brain natriuretic peptide.

a Maximum *N* in quartile, varies slightly with missing covariate data.

**Table 2:** Characteristics of 1331 men without pre-existing CVD or heart failure, by quartile of hsTNT. Figures are mean (SD) unless stated otherwise.

	Quartile of hsTNT (ng/mL)				*P* (trend)	*N*
	1.5–<7.26	≥ 7.26–<11.34	≥ 11.34–<16.92	≥ 16.92–407.10		
	Median = 5.34	Median = 9.23	Median = 13.40	Median = 23.33		
	(*n* = 348)^a^	(*n* = 342)^a^	(*n* = 332)^a^	(*n* = 309)^a^		
Age (years)	76.0 (3.4)	77.9 (4.2)	79.2 (4.7)	81.2 (4.8)	< 0.0001	1331
Manual social class,% (*n*)	48 (166)	46 (154)	45 (148)	44 (134)	0.7	1317
Lives alone,% (*n*)	15 (52)	16 (54)	20 (64)	29 (87)	< 0.0001	1311
Smoker,% (*n*)	4.1 (14)	3.2 (11)	4.0 (13)	3.3 (10)	0.9	1308
Taking statins,% (*n*)	40 (139)	47 (160)	43 (144)	45 (140)	0.3	1331
Taking anti-hypertensives,% (*n*)	44 (152)	55 (187)	50 (167)	65 (201)	< 0.0001	1331
Taking anticoagulants,% (*n*)	2.0 (7)	4.4 (15)	6.0 (20)	11.7 (36)	< 0.0001	1331
Diabetic,% (*n*)	10 (34)	11 (38)	15 (48)	21 (62)	0.001	1331
Alcohol (units per week)	6.9 (7.5)	6.4 (7.7)	6.9 (8.1)	5.3 (7.1)	0.03	1296
BMI (kg/m^2^)	26.5 (3.6)	26.7 (3.6)	27.4 (3.8)	27.4 (4.1)	0.001	1319
Accelerometer wear time (min/day)	857 (65)	864 (69)	851 (63)	847 (69)	0.02	1184
Total activity (counts per day)	187,112 (90,810)	187,241 (99,061)	165,126 (99,061)	122,935 (88,511)	< 0.0001	1184
Steps/day	5500 (2411)	5566 (3047)	4988 (2764)	3767 (2661)	< 0.0001	1184
% time spent sedentary	70.3 (8.5)	70.1 (9.1)	71.7 (9.1)	76.4 (9.7)	< 0.0001	1184
% time light activity	24.3 (6.7)	24.4 (6.5)	23.6 (6.8)	20.5 (7.7)	< 0.0001	1184
% time moderate/vigorous activity	5.4 (3.4)	5.5 (4.2)	4.7 (3.7)	3.2 (3.0)	< 0.0001	1184
Sedentary behaviour (min/day)	601 (78)	605 (85)	609 (83)	644 (81)	< 0.0001	1184
Light activity (min/day)	209 (63)	211 (60)	201 (62)	175 (71)	< 0.0001	1184
Moderate/vigorous activity (min/day)	47 (30)	47 (37)	40 (33)	27 (27)	< 0.0001	1184

Pearson chi square test used for all categorical variables except smoking for which Fisher's exact test was used.

BMI, body mass index.

Light activity, light physical activity.

hsTNT, high sensitivity Troponin T.

a Maximum *N* in quartile, varies slightly with missing covariate data.

**Fig. 1:**
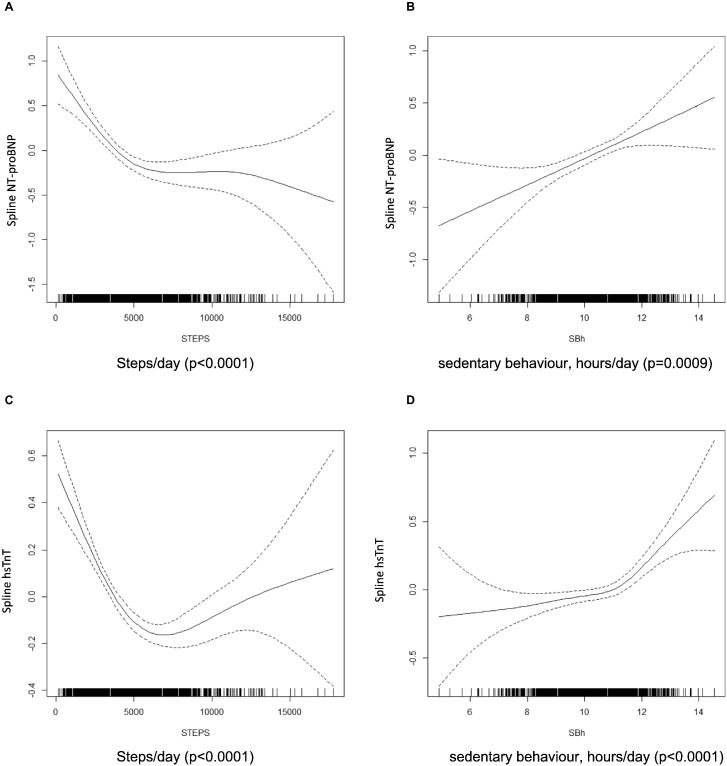
Associations between cardiac biomarkers and physical activity/sedentary behaviour. Relationships between NT-proBNP and steps per day (panel A) and hours per day of sedentary behaviour (panel B) and between hsTnT and steps per day (panel A) and daily hours of sedentary behaviour (panel B). Solid line represents smoothed function from the GAM, with 95% CI (dotted lines), and *p* values are reported. Each model is adjusted for average daily accelerometer wear time, season of wear, hour of blood sampling, region of residence, age, social class, living alone, tobacco, alcohol consumption.

Generalised additive models showed that men who spent more time in physical activity had lower levels of NTproBNP and hsTnT, but relationships were non-linear and significant only at lower levels of physical activity, as illustrated by daily steps in Fig. 1, panels A and C. Total counts, moderate/vigorous activity and light activity each showed a similar pattern (not presented). In contrast, men who spent more time in sedentary behaviour had higher levels of NTproBNP and hsTnT and these associations were linear, as illustrated by daily steps in Fig. 1, panels B and D.

Results from regression models including a spline, are given in Table 3. Higher levels of physical activity were associated with lower levels of NT-proBNP, but significantly only below 150,000 counts per day, 4000 steps per day, 25 min of moderate/vigorous activity per day and 3 h of light activity per day, whereas associations above these knots were not significant (Table 3). For example, in men who undertook < 25 min of moderate/vigorous activity per day, NT-proBNP was 35.7% lower (95% CI − 47.9, − 23.6) for each additional 10 min of moderate/vigorous activity (Table 3, Model 3). For each of the physical activity measures, coefficients above and below the knots were significantly different from each other, *p* < 0.05.

**Table 3:** Cross-sectional associations between physical activity intensity, sedentary behaviour, and markers of cardiac injury.

		ln NT-proBNP % difference	*N* = 1130 (95% CI)		ln hsTnT % difference	*N* = 1130 (95% CI)
**Model 1**						
Additional 10,000 vertical counts/day	At < 150,000 counts/day	**− 7.5**	**(− 10.0, − 5.0)**	At < 150,000 counts/day	**− 4.3**	**(− 5.5, − 3.1)**
	At ≥ 150,000 counts/day	0.2	(− 1.0, 1.3)	At ≥ 150,000 counts/day	0.1	(− 0.4, 0.7)
**Model 2**						
Additional 1000 steps/day	At < 4000 steps/day	**− 28.3**	**(− 37.8, − 18.9)**	At < 6000 steps/day	**− 10.0**	**(− 12.9, − 7.2)**
	At ≥ 4000 steps/day	− 1.9	(− 5.8, 2.0)	At 6000–< 10,000 steps/day	1.5	(− 2.6, 5.6)
				At ≥ 10,000 steps	4.9	(− 1.8, 11.6)
**Model 3**						
Additional 10 min MVPA/day	at < 25 min MVPA/day	**− 35.7**	**(− 47.9, − 23.6)**	At < 50 min MVPA/day	**− 8.4**	**(− 11.1, − 5.6)**
	at ≥ 25 min MVPA/day	− 0.3	(− 3.3, 2.8)	At 50–< 130 min MVPA/day	1.5	(− 1.0, 4.0)
				At ≥ 130 min MVPA/day	2.3	(− 8.3, 12.9)
**Model 4**						
Additional 30 min LPA/day	at < 3 h LPA/day	**− 19.5**	**(− 28.7, − 10.4)**	At < 2.5 h LPA/day	**− 15.5**	**(− 21.8, − 9.1)**
	at ≥ 3 h LPA/day	− 0.2	(− 6.0, 5.5)	At 2.5–< 5 h LPA/day	− 1.4	(− 4.2, 1.5)
				At ≥ 5 h LPA/day	− 2.8	(− 12.0, 6.4)
**Model 5**						
Additional 30 min SB/day		**6.3**	**(3.1, 9.4)**		**3.6**	**(2.1, 5.2)**
**Model 6**						
Additional 10 min MVPA/day	at < 25 min MVPA/day	**− 33.5**	**(− 46.9, − 20.2)**	At < 50 min MVPA/day	**− 6.7**	**(− 10.0, − 3.5)**
	at ≥ 25 min MVPA/day	0.5	(− 3.1, 4.2)	At 50–< 130 min MVPA/day	2.3	(− 0.3, 4.9)
				At ≥ 130 min MVPA/day	3.0	(− 7.6, 13.6)
additional 30 min SB/day		1.7	(− 2.8, 6.2)		2.0	(− 0.2, 4.1)
**Model 7**						
Additional 10 min MVPA/day	at < 25 min MVPA/day	**− 30.6**	**(− 44.3, − 16.9)**	At < 50 min MVPA/day	**− 6.7**	**(− 9.7, − 3.7)**
	at ≥ 25 min MVPA/day	− 0.4	(− 3.5, 2.8)	At 50–< 130 min MVPA/day	1.1	(− 1.4, 3.6)
				At ≥ 130 min MVPA/day	2.8	(− 7.7, 13.4)
Additional 30 min LPA/day	at < 3 h LPA/day	− 8.5	(− 18.7, 1.7)	At < 2.5 h LPA/day	**− 11.5**	**(− 18.1, − 5.0)**
	at ≥ 3 h LPA/day	1.1	(− 4.8, 7.0)	At 2.5–< 5 h LPA/day	0.4	(− 2.6, 3.4)
				At ≥ 5 h LPA/day	− 2.9	(− 12.1, 6.2)

Estimates provided are percentage differences (95% CI) in biomarker levels for specified increases in physical activity or sedentary time parameter, derived from linear regression analyses.

Bold text indicates differences which are statistically significant (*p* < 0.05).

All coefficients adjusted for average daily accelerometer wear time, season of wear, hour of blood sampling, region of residence, age, social class, living alone, tobacco, alcohol consumption.

SB, sedentary behaviour, LPA, light physical activity, MVPA, moderate and vigorous physical activity.

NT-proBNP, N-terminal pro-brain natriuretic peptide, hsTnT, high sensitivity Troponin.

Similarly, higher levels of physical activity were associated with lower levels of hsTnT, but associations were significant only below the daily levels of counts, steps, moderate/vigorous activity and light activity indicated in Table 3. For example, in men who undertook < 6000 steps per day, hsTnT was 10.0% lower (95% CI − 12.9, − 7.2) for each additional 1000 steps, but there was no significant association above 6000 steps per day (Table 3, Model 2).

Associations between sedentary behaviour and cardiac injury markers were linear and in the opposite direction. For each additional 30 min of sedentary behaviour, NT-proBNP levels were 6.3% higher (95% CI 3.1, 9.4), and hsTnT 3.6% higher (95% CI 2.1, 5.2) (Table 3 Model 5). Associations between NT-proBNP or hsTnT and moderate/vigorous activity were slightly reduced after adjusting for sedentary behaviour (Table 3, Model 6), whilst associations with sedentary behaviour were abolished. Associations between NT-proBNP or hsTnT and moderate/vigorous activity were slightly reduced after adjusting for light activity and although the association between NTproBNP and light activity was abolished, that between hsTnT and light activity persisted albeit reduced in magnitude by about 40%. Adjusting models for BMI made little difference to results.

After excluding 191 men with NTproBNP levels > 400 pg/mL, associations between physical activity measures and NTproBNP or hsTnT were reduced in magnitude but remained significant except for associations between NTproBNP and light activity or sedentary behaviour (details in Supplementary file). Post hoc analyses showed that men with hypertension had higher levels of NTproBNP and hsTnT than men with normal blood pressure, and regression models stratified by hypertensive status showed that associations between NTproBNP and physical activity were present only in men with hypertension (see Supplementary file). Associations between hsTnT and physical activity did not differ by hypertensive status.

## 4 Discussion

In our study of British men from a seldom studied older age group (71–90 years), we found that higher levels of physical activity were associated with lower levels of NT-proBNP and hsTnT, but the relationships were non-linear and associations only seen at low to moderate levels of activity. The shape of the relationship was similar for each of the activity measures we investigated, total counts, steps, moderate/vigorous activity and light activity. This non-linearity is consistent with evidence that suggests that at most levels physical activity shows beneficial associations with cardiac biomarkers, structure and function, but at high levels physical activity can increase the release of BNP and TnT, together with risks of CVD [22]. Sedentary behaviour was related to NT-proBNP and hsTnT in the opposite direction; higher levels of sedentary behaviour were associated with higher levels of NT-proBNP and hsTnT, and these associations were linear.

Previously in this cohort when the men were aged 60–79 years, higher self-reported physical activity was associated with lower NT-proBNP [23] and there was some evidence that the beneficial effects of activity on CHD and all-cause mortality were mediated via a beneficial effect on NT-proBNP level. In addition, a 1 standard deviation (SD) increase in log_e_ NT-proBNP has been associated with an adjusted hazard ratio (HR) for major CVD events of 1.49 (95% CI 1.33, 1.65) and a 1SD increase in log_e_ hsTnT with an adjusted HR of 1.37 [24]. In our analyses, among men taking < 4000 steps per day, an additional 1000 steps per day was associated with a decrease in log_e_ NT-proBNP of 0.283, one fifth of 1SD (1.36), and extrapolating from earlier data might therefore be associated with a HR of 0.9 for major CVD events. This is plausible given that an Australian study recently reported that higher daily steps was associated with a lower risk of all-cause mortality, with an adjusted hazard ratio of a similar magnitude; 0.94 (95% CI 0.90, 0.98) for each additional 1000 steps [25], but how much the benefits of physical activity are mediated via cardiac biomarkers requires further exploration.

In our study, the associations between sedentary behaviour and NT-proBNP or hsTnT did not persist when moderate/vigorous activity was included in the model, whereas associations with moderate/vigorous activity were little affected, suggesting that moderate/vigorous activity is the important factor for these biomarkers, with light activity also playing a role for hsTnT. Previous studies have shown obesity to be related to abnormal cardiac remodeling [26] and impaired myocardial contractile function [26] as well as higher hsTnT levels [27], [28]. The pathways are not yet fully defined but adjusting relationships between physical activity/sedentary behaviour and hsTnT for BMI in our data did not greatly affect coefficients, suggesting that any effect of physical activity may be at least partially independent of BMI [29]. Adjusting for BMI did not change relationships between physical activity/sedentary behaviour and NT-proBNP in our study, again suggesting a possible independent role for physical activity. Excluding men with high NT-proBNP levels (> 400 pg/L) indicating potential heart failure reduced the magnitude of associations but did not change the overall findings of our study. Post hoc analysis showed that the associations between physical activity measures and NTproBNP were present in men with hypertension but not in men with normal blood pressure. Possibly in the hypertensive group men who have higher NTproBNP are those with early symptoms of diagnosed or undiagnosed heart failure who get out of breath and therefore are less physically active, and/or the reduced sample size of men with normal blood pressure (*n* = 357) may limit power to detect associations. Given this was a post hoc analysis, this finding requires further study.

## 5 Strengths and limitations

Whilst the main limitation of our study is the cross-sectional design which means we cannot infer causality, a major strength is the use of objectively measured physical activity, which allowed us to investigate in detail the shape of the relationship between activity and biomarkers of cardiac injury, and to explore activity of different intensities including sedentary behaviour. Participants were highly compliant with the accelerometer wear protocol; 96% of men provided the ≥ 5 days data needed to predict habitual physical activity/sedentary behaviour [30]. Sedentary behaviour usually refers to time spent sitting, and we recognise that the Actigraph accelerometer does not differentiate well between sitting and standing during periods of < 100 cpm. However, mean count levels for these minutes were < 10 cpm indicating that these minutes were very sedentary. Our study utilises data from an understudied older age group, and has the advantage of being population based rather than confined to an at risk group, but results may not be generalizable to younger age-groups, women or non-European ethnic groups. The study sample remains socially representative of the UK [31] although men who participated in our study were healthier (slightly younger and slightly lower BMI) than those who did not. Given the large range in physical activity and levels of biomarkers among participants we would not expect associations between activity and biomarkers to be influenced by this selection effect.

## 6 Conclusions

Our study identified non-linear relationships between accelerometer measured physical activity and both NT-proBNP and hsTnT, such that higher levels of total activity, moderate/vigorous activity and light activity were associated with lower levels of biomarkers at low/moderate levels of activity. Higher levels of sedentary behaviour were associated (linearly) with higher levels of biomarkers, but not independently of moderate/vigorous activity. Physical activity may be more important in protecting against subclinical cardiac ischaemia or myocardial stress and injury in less active individuals and this might be part of the mechanism by which physical activity protects against a deterioration in cardiac health which leads to clinical heart failure and CVD. However, confirmation in other prospective studies is needed.

## Funding

This work was supported by the British Heart Foundation [PG/13/86/30546, PG09/024, RG/08/013/25942] and the National Institute for Health Research [Post-Doctoral Fellowship 2010–03–023]. The funders had no role in the design and conduct of the study; collection, management, analysis, and interpretation of the data; preparation, review, or approval of the manuscript; and decision to submit the manuscript for publication.

## Conflict of interest

The authors report no relationships that could be construed as a conflict of interest.

## Appendix A Supplementary data

Supplementary material Image 1

## References

[1] Ponikowski P., Voors A.A., Anker S.D., Bueno H., Cleland J.G., Coats A.J., Falk V., Gonzalez-Juanatey J.R., Harjola V.P., Jankowska E.A., Jessup M., Linde C., Nihoyannopoulos P., Parissis J.T., Pieske B., Riley J.P., Rosano G.M., Ruilope L.M., Ruschitzka F., Rutten F.H., van der Meer P., Authors/Task Force M (2016). 2016 ESC guidelines for the diagnosis and treatment of acute and chronic heart failure: the task force for the diagnosis and treatment of acute and chronic heart failure of the European Society of Cardiology (ESC) developed with the special contribution of the Heart Failure Association (HFA) of the ESC. Eur. Heart J..

[2] (NICE) NIFHACE (2014). Myocardial Infarction (Acute): Early Rule Out Using High-sensitivity Troponin Tests (Elecsys Troponin T High-sensitive, ARCHITECT STAT High Sensitive Troponin-I and AccuTnI + 3 Assays).

[3] de Filippi C.R., de Lemos J.A., Christenson R.H., Gottdiener J.S., Kop W.J., Zhan M., Seliger S.L. (2010). Association of serial measures of cardiac troponin T using a sensitive assay with incident heart failure and cardiovascular mortality in older adults. JAMA.

[4] Daniels L.B., Clopton P., de Filippi C.R., Sanchez O.A., Bahrami H., Lima J.A., Tracy R.P., Siscovick D., Bertoni A.G., Greenland P., Cushman M., Maisel A.S., Criqui M.H. (2015). Serial measurement of N-terminal pro-B-type natriuretic peptide and cardiac troponin T for cardiovascular disease risk assessment in the Multi-Ethnic Study of Atherosclerosis (MESA). Am. Heart J..

[5] Oluleye O.W., Folsom A.R., Nambi V., Lutsey P.L., Ballantyne C.M. (2013). Troponin T, B-type natriuretic peptide, C-reactive protein, and cause-specific mortality. Ann. Epidemiol..

[6] Wannamethee S.G., Welsh P., Whincup P.H., Lennon L., Papacosta O., Sattar N. (2014). N-terminal pro brain natriuretic peptide but not copeptin improves prediction of heart failure over other routine clinical risk parameters in older men with and without cardiovascular disease: population-based study. Eur. J. Heart Fail..

[7] Natriuretic Peptides Studies C (2016). Natriuretic peptides and integrated risk assessment for cardiovascular disease: an individual-participant-data meta-analysis. Lancet Diabetes Endocrinol..

[8] Echouffo-Tcheugui J.B., Butler J., Yancy C.W., Fonarow G.C. (2015). Association of physical activity or fitness with incident heart failure: a systematic review and meta-analysis. Circ. Heart Fail..

[9] Pandey A., Garg S., Khunger M., Darden D., Ayers C., Kumbhani D.J., Mayo H.G., de Lemos J.A., Berry J.D. (2015). Dose-response relationship between physical activity and risk of heart failure: a meta-analysis. Circulation.

[10] Nayor M., Vasan R.S. (2015). Preventing heart failure: the role of physical activity. Curr. Opin. Cardiol..

[11] Hegde S.M., Goncalves A., Claggett B., Evenson K.R., Cheng S., Shah A.M., Folsom A.R., Solomon S.D. (2016). Cardiac structure and function and leisure-time physical activity in the elderly: the Atherosclerosis Risk in Communities Study. Eur. Heart J..

[12] Young D.R., Reynolds K., Sidell M., Brar S., Ghai N.R., Sternfeld B., Jacobsen S.J., Slezak J.M., Caan B., Quinn V.P. (2014). Effects of physical activity and sedentary time on the risk of heart failure. Circ. Heart Fail..

[13] Klenk J., Denkinger M., Nikolaus T., Peter R., Rothenbacher D., Koenig W. (2013). Association of objectively measured physical activity with established and novel cardiovascular biomarkers in elderly subjects: every step counts. J. Epidemiol. Community Health.

[14] de Filippi C.R., de Lemos J.A., Tkaczuk A.T., Christenson R.H., Carnethon M.R., Siscovick D.S., Gottdiener J.S., Seliger S.L. (2012). Physical activity, change in biomarkers of myocardial stress and injury, and subsequent heart failure risk in older adults. J. Am. Coll. Cardiol..

[15] Srivastava P.K., Pradhan A.D., Cook N.R., Ridker P.M., Everett B.M. (2016). Impact of modifiable risk factors on B-type natriuretic peptide and cardiac troponin T concentrations. Am. J. Cardiol..

[16] Jefferis B.J., Sartini C., Ash S., Lennon L.T., Wannamethee S.G., Whincup P.H. (2016). Validity of questionnaire-based assessment of sedentary behaviour and physical activity in a population-based cohort of older men; comparisons with objectively measured physical activity data. Int. J. Behav. Nutr. Phys. Act..

[17] Jefferis B.J., Sartini C., Lee I.M., Choi M., Amuzu A., Gutierrez C., Casas J.P., Ash S., Lennnon L.T., Wannamethee S.G., Whincup P.H. (2014). Adherence to physical activity guidelines in older adults, using objectively measured physical activity in a population-based study. BMC Public Health.

[18] Choi L., Liu Z., Matthews C.E., Buchowski M.S. (2011). Physical Activity: Process Physical Activity Accelerometer Data (0.1–1).

[19] Copeland J.L., Esliger D.W. (2009). Accelerometer assessment of physical activity in active, healthy older adults. J. Aging Phys. Act..

[20] Giannitsis E., Kurz K., Hallermayer K., Jarausch J., Jaffe A.S., Katus H.A. (2010). Analytical validation of a high-sensitivity cardiac troponin T assay. Clin. Chem..

[21] Prontera C., Emdin M., Zucchelli G.C., Ripoli A., Passino C., Clerico A. (2004). Analytical performance and diagnostic accuracy of a fully-automated electrochemiluminescent assay for the N-terminal fragment of the pro-peptide of brain natriuretic peptide in patients with cardiomyopathy: comparison with immunoradiometric assay methods for brain natriuretic peptide and atrial natriuretic peptide. Clin. Chem. Lab. Med..

[22] O'Keefe J.H., Patil H.R., Lavie C.J., Magalski A., Vogel R.A., McCullough P.A. (2012). Potential adverse cardiovascular effects from excessive endurance exercise. Mayo Clin. Proc..

[23] Jefferis B.J., Whincup P.H., Lennon L.T., Papacosta O., Goya Wannamethee S. (2014). Physical activity in older men: longitudinal associations with inflammatory and hemostatic biomarkers, N-terminal pro-brain natriuretic peptide, and onset of coronary heart disease and mortality. J. Am. Geriatr. Soc..

[24] Welsh P., Hart C., Papacosta O., Preiss D., McConnachie A., Murray H., Ramsay S., Upton M., Watt G., Whincup P., Wannamethee G., Sattar N. (2016). Prediction of cardiovascular disease risk by cardiac biomarkers in 2 United Kingdom cohort studies: does utility depend on risk thresholds for treatment?. Hypertension.

[25] Dwyer T., Pezic A., Sun C., Cochrane J., Venn A., Srikanth V., Jones G., Shook R.P., Sui X., Ortaglia A., Blair S., Ponsonby A.L. (2015). Objectively measured daily steps and subsequent long term all-cause mortality: the Tasped Prospective Cohort Study. PLoS One.

[26] Abel E.D., Litwin S.E., Sweeney G. (2008). Cardiac remodeling in obesity. Physiol. Rev..

[27] McEvoy J.W., Lazo M., Chen Y., Shen L., Nambi V., Hoogeveen R.C., Ballantyne C.M., Blumenthal R.S., Coresh J., Selvin E. (2015). Patterns and determinants of temporal change in high-sensitivity cardiac troponin-T: the Atherosclerosis Risk in Communities Cohort Study. Int. J. Cardiol..

[28] Pervanidou P., Akalestos A., Bastaki D., Apostolakou F., Papassotiriou I., Chrousos G. (2013). Increased circulating high-sensitivity troponin T concentrations in children and adolescents with obesity and the metabolic syndrome: a marker for early cardiac damage?. Metabolism.

[29] Ndumele C.E., Matsushita K., Sang Y., Lazo M., Agarwal S.K., Nambi V., Deswal A., Blumenthal R.S., Ballantyne C.M., Coresh J., Selvin E. (2016). N-terminal pro-brain natriuretic peptide and heart failure risk among individuals with and without obesity: the Atherosclerosis Risk in Communities (ARIC) Study. Circulation.

[30] Hart T.L., Swartz A.M., Cashin S.E., Strath S.J. (2011). How many days of monitoring predict physical activity and sedentary behaviour in older adults?. Int. J. Behav. Nutr. Phys. Act..

[31] Lennon L.T., Ramsay S.E., Papacosta O., Shaper A.G., Wannamethee S.G., Whincup P.H. (2015). Cohort profile update: the British Regional Heart Study 1978–2014: 35 years follow-up of cardiovascular disease and ageing. Int. J. Epidemiol..

