# Supplementary material for: Objectively measured physical activity and cardiac biomarkers: A cross sectional population based study in older men
Source: Int J Cardiol. 2018 Mar 1;254:322–7. doi: 10.1016/j.ijcard.2017.11.003 (PMC5958950; doi:10.1016/j.ijcard.2017.11.003)
Supplement: Supplementary file 1 — Supplementary material [file mmc1.docx]

**Results (Supplementary)**

*Excluding men with high NTproBNP levels*

After excluding men with NTproBNP levels >400pg/ml associations between physical activity measures (total counts, steps, moderate/vigorous activity, light activity, sedentary behaviour) and NTproBNP or hsTnT were reduced in magnitude, by 50-58% for NTproBNP and by 23-40% for hsTnT, but were in the same direction, with significance maintained except for associations between NTproBNP and light activity or sedentary behaviour which were reduced by a larger amount and abolished. In models investigating associations between moderate/vigorous activity and biomarkers but including adjustment for sedentary behaviour, NTproBNP was 18.8% lower (95% CI -31.5, -6.2) for every additional 10 minutes of moderate/vigorous activity, and hsTnT was 5.5% lower (95% CI -9.0, -2.2) compared with 33.5% and 6.7% lower respectively before exclusion (Table 3 model 6), among men undertaking less than 25 minutes of moderate/vigorous activity per day.

*Post hoc analysis stratified by hypertensive status*

Men with hypertension had higher levels of NTproBNP and hsTnT than men with normal blood pressure, and were less likely to smoke, take statins, anticoagulants, have diabetes, and had a higher mean BMI and consumed more alcohol (Supplementary Table 1). Hypertensive men spent a higher percentage of their time being sedentary and lower percentage of time in light and moderate/vigorous activity (Supplementary Table 1).

Regression models examining the associations between physical activity measures and NTproBNP showed significant interactions between hypertensive status and activity, and therefore models were run stratifying by hypertensive status. Generalised additive models showed that in men with normal blood pressure the relationships between physical activity or sedentary behaviour variables and NTproBNP were approximately linear or null (Supplementary Table 2). In hypertensive men the associations between activity variables and NTproBNP were similar in shape to the associations in the whole sample of men, i.e. non-linear for total counts, steps, moderate/vigorous activity and light activity and linear for sedentary behaviour. Slightly different cut points were used for the splines. Higher levels of physical activity were associated with lower levels of NTproBNP, but associations were significant only below the daily levels of counts, steps, moderate/vigorous activity and light activity indicated in Supplementary Table 2. For example, in men who undertook less than 5,000 steps per day, NTproBNP was 24.0% lower (95% CI -34.9, -18.1) for each additional 1,000 steps, but there was no significant association above 5,000 steps per day (Supplementary Table 2, model 2).

The associations between physical activity measures and hsTnT were not influenced by hypertensive status (data not shown).

**Supplementary Table 1. Cardiac Injury biomarkers and characteristics of 1331 men without pre-existing CVD or heart failure, by hypertension status. Values are mean (SD) unless stated otherwise.**

|  | **normotensive**  **n=425^b^** | **hypertensive^a^**  **n=906^b^** | **p** | **N** |
| --- | --- | --- | --- | --- |
| NT-proBNP (pg/mL)^c^ | 106 (4) | 137 (4) | 0.001 | 1331 |
| hsTnT (ng/L)^c^ | 9.5 (2.0) | 11.4 (2.0) | <0.0001 | 1331 |
| Age (years) | 78.2 (4.7) | 78.6 (4.6) | 0.2 | 1331 |
| Manual Social class,% (n) | 45 (189) | 46 (413) | 0.8 | 1317 |
| Lives alone,% (n) | 19 (78) | 20 (179) | 0.6 | 1311 |
| Smoker,% (n) | 6.0 (25) | 2.6 (23) | 0.003 | 1308 |
| Taking statins,% (n) | 23 (98) | 54 (485) | <0.0001 | 1331 |
| Taking anticoagulants,% (n) | 1.7 (7) | 7.8 (71) | <0.0001 | 1331 |
| Diabetic,% (n) | 8.2 (35) | 16.2 (147) | <0.0001 | 1331 |
| Alcohol (units per week) | 6.3 (7.6) | 6.4 (7.7) | 0.8 | 1296 |
| BMI (kg/m^2^) | 26.4 (3.6) | 27.2 (3.8) | <0.0001 | 1319 |
| accelerometer wear time (mins/day) | 864 (71) | 851 (64) | 0.002 | 1184 |
| total activity (counts per day) | 183,555 (105,975) | 159,819 (97,034) | <0.0001 | 1184 |
| steps / day | 5336 (2833) | 4848 (2794) | 0.005 | 1184 |
| % time SB | 70.7 (9.4) | 72.6 (9.3) | 0.001 | 1184 |
| % time LPA | 24.1 (7.2) | 22.9 (7.0) | 0.006 | 1184 |
| % time MVPA | 5.2 (3.8) | 4.5 (3.7) | 0.006 | 1184 |
| SB (mins/day) | 609 (84) | 616 (83) | 0.2 | 1184 |
| LPA (mins/day) | 210 (68) | 196 (64) | 0.001 | 1184 |
| MVPA (mins/day) | 45 (34) | 39 (32) | 0.002 | 1184 |

^a^hypertension defined as systolic blood pressure≥160mmHg or diastolic blood pressure ≥90mmHg or taking anti-hypertensive medication

^b^ maximum N in group, varies slightly with missing covariate data

^c^geometric means given

Pearson chi square test used for all categorical variables except smoking for which Fisher’s exact test was used

NT-proBNP, N-terminal pro-brain natriuretic peptide

hsTNT, high sensitivity Troponin T

BMI, body mass index

SB, sedentary behaviour

LPA, light physical activity

MVPA, moderate and vigorous physical activity

**Supplementary Table 2. Cross-sectional associations between physical activity intensity, sedentary behaviour, and NT-proBNP, by hypertension status.**

|  | **normotensive** | |  |  | **hypertensive** | |
| --- | --- | --- | --- | --- | --- | --- |
|  | **ln NT-proBNP** | **N=357** |  |  | **ln NT-proBNP** | **N=773** |
|  | **% difference** | **(95% CI)** |  |  | **% difference** | **(95% CI)** |
| **Model 1** |  |  |  |  |  |  |
| additional 10,000 counts/day | -0.07 | (-1.52, 1.38) |  | at <150,000 counts/day | **-9.2** | **(-12.1, -6.2)** |
|  |  |  |  | at ≥150,000 counts/day | 0.2 | (-1.3, 1.6) |
|  |  |  |  |  |  |  |
| **Model 2** |  |  |  |  |  |  |
| additional 1000 steps/day | -1.77 | (-7.15, 3.61) |  | at <5,000 steps/day | **-26.5** | **(-34.9,-18.1)** |
|  |  |  |  | at ≥5,000 steps/day | -0.2 | (-5.7, 5.4) |
|  |  |  |  |  |  |  |
| **Model 3** |  |  |  |  |  |  |
| additional 10 mins MVPA/day | 0.21 | (-4.14, 4.55) |  | at <40 mins MVPA/day | **-24.0** | **(-32.4, -15.7)** |
|  |  |  |  | at ≥40 mins MVPA/day | 0.9 | (-3.7, 5.4) |
|  |  |  |  |  |  |  |
| **Model 4** |  |  |  |  |  |  |
| additional 30 mins LPA/day | 2.05 | (-4.82, 8.91) |  | at <3 hrs LPA/day | **-23.0** | **(-33.6, -12.5)** |
|  |  |  |  | at ≥3 hrs LPA/day | -2.4 | (-10.0, 5.1) |
|  |  |  |  |  |  |  |
| **Model 5** |  |  |  |  |  |  |
| additional 30 mins SB/day | -1.36 | (-6.74, 4.02) |  |  | **9.4** | **(5.5, 13.2)** |
|  |  |  |  |  |  |  |
|  |  |  |  |  |  |  |
| **Model 6** |  |  |  |  |  |  |
| additional 10 mins MVPA/day | -0.98 | (-6.86, 4.90) |  | at <40 mins MVPA/day | **-19.9** | **(-29.7, -10.0)** |
|  |  |  |  | at ≥40 mins MVPA/day | 2.7 | (-2.4, 7.9) |
|  |  |  |  |  |  |  |
| additional 30 mins SB/day | -2.18 | (-9.47, (5.11) |  |  | 4.5 | (-1.1, 10.1) |
|  |  |  |  |  |  |  |
|  |  |  |  |  |  |  |
| **Model 7** |  |  |  |  |  |  |
| additional 10 mins MVPA/day | -0.25 | (-4.86, 4.36) |  | at <40 mins MVPA/day | **-19.3** | **(-28.6, -9.9)** |
|  |  |  |  | at ≥40 mins MVPA/day | 0.6 | (-4.0, 5.3) |
|  |  |  |  |  |  |  |
| additional 30 mins LPA/day | 2.18 | (-5.11, 9.47) |  | at <3 hrs LPA/day | **-13.1** | **(-24.5, -1.6)** |
|  |  |  |  | at ≥3 hrs LPA/day | 0.1 | (-7.6, 7.9) |
|  |  |  |  |  |  |  |

Estimates provided are percentage differences (95% CI) in NT-proBNP for specified increases in physical activity or sedentary time parameter, derived from linear regression analyses

Bold text indicates differences which are statistically significant (p < 0.05)

All coefficients adjusted for average daily accelerometer wear time, season of wear, hour of blood sampling, region of residence, age, social class, living alone, tobacco, alcohol consumption

MVPA, moderate and vigorous physical activity

LPA, light physical activity

SB, sedentary behaviour

NT-proBNP, N-terminal pro-brain natriuretic peptide
